# Hemorrhagic Sudden Onset of Spinal Epidural Angiolipoma

**DOI:** 10.1155/2018/5231931

**Published:** 2018-07-02

**Authors:** Kiyotaka Horiuchi, Tsuyoshi Yamada, Kenichiro Sakai, Atsushi Okawa, Yoshiyasu Arai

**Affiliations:** ^1^Department of Orthopaedic Surgery, Saiseikai Kawaguchi General Hospital, Saitama 332-8558, Japan; ^2^Department of Orthopaedic Surgery, Graduate School, Tokyo Medical and Dental University, 1-5-45 Yushima, Bunkyo-ku, Tokyo 113-8510, Japan

## Abstract

Angiolipomas are relatively rare benign tumors. Spinal angiolipomas that generally induce slow progressive cord compression are most commonly found in the thoracic region. A 49-year-old female with obesity presented with a 1-week history of progressively worsening back pain, paresthesia of lower limbs, and gait disturbance. When thoracic magnetic resonance imaging (MRI) revealed a dorsal epidural mass at the Th5–Th8 level, the patient underwent a laminectomy for gross total excision of the lesion. Both mature fatty tissue and abnormal proliferating vascular elements with thin or expanded walls were observed in the resected tumor. Nonfiltrating spinal angiolipoma was diagnosed and confirmed by pathology. After the operation, sensory loss, numbness, and gait disturbance were improved following the disappearing severe back pain. Following examinations indicated the absence of recurrence within 1 year. The angiolipomas of the spine are rare causes of spinal cord compression that generally induce slow progressive cord compression, but sudden onset or rapid worsening of neurological deterioration is observed in hemorrhagic spinal angiolipoma.

## 1. Introduction

Angiolipomas are relatively rare benign tumors. It is reported that angiolipomas accounted for 0.04% to 1.2% of all tumors of the spine with vascular and fatty histological features [1]. Spinal angiolipomas that generally induce slow progressive cord compression are most commonly found in the thoracic region and have a high signal in contrast-enhanced fat-saturated T1-weighted imaging. However, the predominance of either vascular or fatty components inside this tumor could alter the expected results on magnetic resonance imaging (MRI) with fat-suppression sequences [2].

Owing to its rare occurrence, this tumor is often misdiagnosed as epidural hematoma before surgical excision of the lesion. Although they most commonly have an insidious course, the acute presentation was reported in a few cases. Here, the authors described one case of a hemorrhagic spinal angiolipoma with sudden onset.

## 2. Case Presentation

A 49-year-old female with obesity (body mass index: 31.1 kg/m^2^) presented with a 1-week history of progressively worsening back pain, paresthesia of the lower limbs, and gait disturbance. Moderate muscle weakness in the lower limbs, a superficial hypesthesia below the T5 level, and a dorsal cord disorder was noted at the first physical examination.

**Figure 1:**
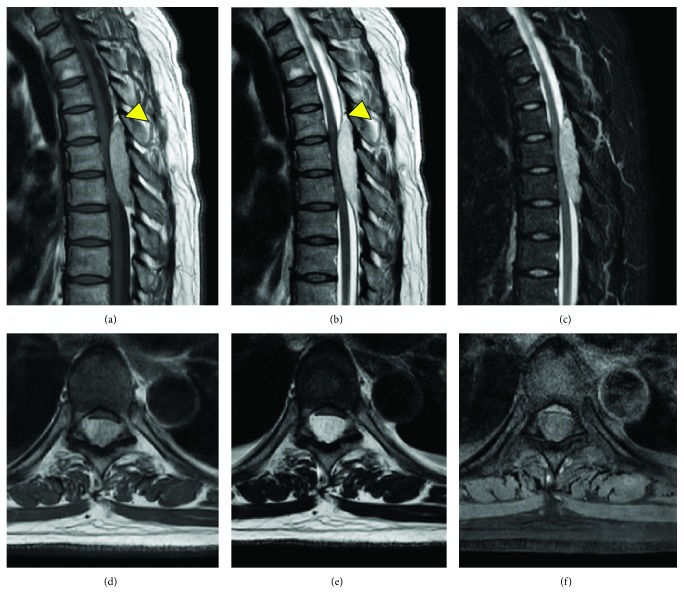
Preoperative thoracic MRI. A heterogeneous epidural posterior mass compressing the thoracic cord between Th5 and Th8 was revealed at admission. Sagittal view of the heterogeneous isointense mass of the T1-weighted image (a), heterogeneous hyperintense mass of the T2-weighted image (b), and enhanced mass of the fat-suppression image by short-T1 inversion recovery (STIR) (c). Axial view of the epidural mass of the T1-weighted image (d), T2-weighted image (e), and STIR image (f). This tumor had lipomatosis in its upper segment (arrowhead).

**Figure 2:**
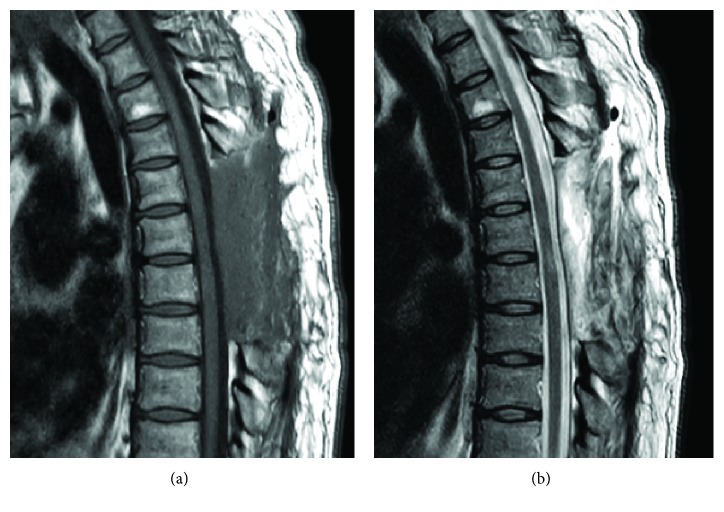
Postoperative thoracic MRI (seven days after surgery). Postoperative MRI showed the complete resection of the tumors. Sagittal view of the T1-weighted image (a) and T2-weighted image (b).

Laboratory investigations and plain radiography revealed no abnormality. MRI showed a dorsally located epidural lesion (Th5–Th8) which seemed to be a heterogeneous mass that was isointense on T1-weighted imaging and slightly hyperintense on T2-weighted imaging (Figure 1). These clinical courses and radiological findings suggested epidural hematoma. An emergent surgical excision of the lesion was performed. When Th5–8 laminectomy was performed, the posterior epidural space was filled with a fatty, highly vascular brown-pink mass. A small mass of epidural fat (lipomatosis) was encountered at both the upper and lower end of the lesion. En bloc resection of the tumor was difficult, and the tumor was totally removed piecemeal. Adhesions between these tumors and dura were slight. Intraoperative blood loss reached 2000 mL despite repeating hemostasis by electrocoagulation. The complete resection of these extremely hemorrhagic adipose components made compressive dura matter swollen (Figure 2).

**Figure 3:**
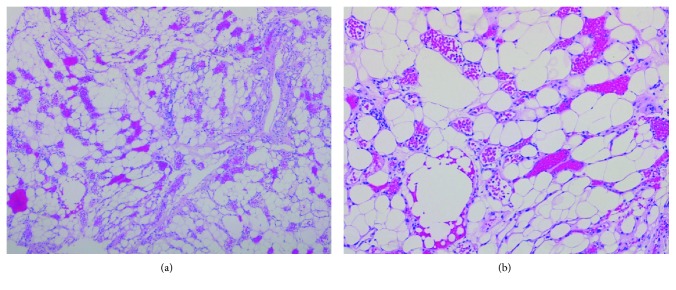
Histopathological analysis of the resected tumors. The spinal angiolipoma consists of both mature fatty tissue and abnormal proliferating vascular elements with thin or expanded walls, which usually contain fibrin thrombi (hematoxylin and eosin stained; magnification: (a) ×40 and (b) ×100).

Both mature fatty tissue and abnormal proliferating vascular elements with thin or expanded walls were observed in the resected tumor. Intratumoral thrombosis was also partially found. Nonfiltrating spinal angiolipoma was diagnosed and confirmed by pathology (Figure 3). After the operation, sensory loss, numbness, and gait disturbance were improved. Her Japanese Orthopaedic Association (JOA) score for thoracic myelopathy recovered from a preoperative 4.5 points to 9.5 points out of 11 points. Following examinations indicated the absence of recurrence within 1 year.

## 3. Discussion

Angiolipomas which are composed of fatty tissue and vascular elements are benign tumors and usually present with a slowly progressive course of neurological deterioration. The origin and pathogenesis of angiolipomas remain unknown while pluripotential mesenchymal stem cells [3], congenital malformation, benign hamartoma [4], and primitive mesenchyme [5] could be associated with its specific entity. It is reported that spinal angiolipomas are predominantly found in the dorsal part of the spinal cord, and the appearance of the sensory disorders is ahead of the motor deficits [6]. Akhaddar et al. presented the first case of intraspinal bleeding from an epidural angiolipoma producing hyperacute paraplegia and simulating an extradural hematoma in 2008 [7]. Almost all the reported spinal angiolipomas with bleeding were located in the midthoracic region, and caused not only back pain or paresthesia but was also followed by paraplegia within a few minutes to a 1-week [7–10].

Likewise, a rapid neurological deterioration was observed in the current case that is why we had initially diagnosed it as spinal epidural hemorrhage. Sudden onset or worsening of neurological findings occurred when there is a rapid increase in tumor size of angiolipomas due to intratumoral thrombosis, massive acute hemorrhage, or steal phenomenon [5, 7–9]. Vigorous exercise can contribute to augment blood flow size in the tumor to produce epidural bleeding [7]. The findings of pathology, including plentiful blood vessels and intratumoral thrombosis, supported this progressive clinical course in the present case. The predominance of these vascular components inside the tumor could attributed to the sudden onset and massive hemorrhage.

Angiolipomas used to be categorized into two subtypes: noninfiltrating and infiltrating. Recently, the two types were regarded as basically the same, and the bone damage of the infiltrating type could be caused by the tumor's expansive oppression. Si et al. established the new angiolipoma classification as Type I (intraspinal: Type IA without lipomatosis and Type IB with lipomatosis in its upper and/or lower segments) and Type II (dumbbell shaped) [6]. They did not regard angiolipomas as protean, and the signals of angiolipomas in the MRI examination were basically the same. MRI typically shows an iso- to hyperintense mass on T1-weighted images and hyperintense without flow voids on T2-weighted images, while the variable vascular and adipose elements of the tumor often shows significant heterogeneity in the MRI studies [2, 11]. In our case, in sagittal T1-weighted images, the tumor's signal was inhomogeneous with an isointense signal present in the center part and a high signal present in the upper part. In T2-weighted images, the signal was slightly high in the center part and hyperintense in the upper part. We retrospectively found that this tumor had lipomatosis in its upper segment (Type IB). The present case was consistent with the fact that overweight people were more likely to get the Type IB [6].

Differential diagnosis must include some epidural tumors and spinal epidural hematomas especially in the acute presentation. Heterogeneous hyperintensity to the spinal cord with focal hypointensity on T2-weighted imaging should suggest the diagnosis of acute spinal epidural hematoma [10]. Furthermore, all the cases of acute spinal epidural hematomas which needed emergent surgery in our institute (8 cases, from 2010 to 2016) showed a hypointense mass on T1-weighted imaging while the intensity on T2-weighted images seemed variable. On the other hand, as for angiolipoma with bleeding, MRI seems to show an inhomogenous isointense mass on T1-weighted images. T1-weighted images could provide useful information for differential diagnosis of acute spinal cord compression. If MRI and a rapid clinical course suggest a substantial vascular tumor, angiography and embolization should be performed before surgery to facilitate surgical excision [12]. The clinical outcomes of Type IB are slightly worse than Type IA because of the spinal cord compression by the remaining lipomatosis, although malformations and recurrence are exceptional in spinal angiolipomas [6]. Total resection of the angiolipoma, including this upper lipomatosis, could be achieved in the present case (Figure 2) and the prognosis after surgery was good without the adjuvant radiation therapy.

The angiolipomas of the spine are rare causes of spinal cord compression that generally induce slow progressive cord compression, but sudden onset or rapid worsening of neurological deterioration is observed in hemorrhagic spinal angiolipoma.
